# Atopic eczema and fracture risk in adults: A population-based cohort study

**DOI:** 10.1016/j.jaci.2019.09.015

**Published:** 2020-02

**Authors:** Katherine E. Lowe, Kathryn E. Mansfield, Antonella Delmestri, Liam Smeeth, Amanda Roberts, Katrina Abuabara, Daniel Prieto-Alhambra, Sinéad M. Langan

**Affiliations:** a Department of Non-Communicable Disease Epidemiology, London School of Hygiene and Tropical Medicine, London, United Kingdom; b Centre for Statistics in Medicine, Nuffield Department of Orthopaedics, Rheumatology, and Musculoskeletal Sciences, University of Oxford, Oxford, United Kingdom; c Department of Dermatology, University of California–San Francisco, San Francisco, Calif; d Health Data Research UK, London, United Kingdom; e Department of Epidemiology, Colorado School of Public Health, University of Colorado, Anschutz Medical Campus, Aurora, Colo; f Nottingham Support Group for Carers of Children with Eczema, Nottingham, United Kingdom

**Keywords:** Atopic eczema, fracture, osteoporosis, population based, severity, BMI, Body mass index, CPRD, Clinical Practice Research Datalink, HES, Hospital Episode Statistics, HR, Hazard ratio, IMD, Index of Multiple Deprivation, UK, United Kingdom

## Abstract

**Background:**

Limited evidence suggests increased fracture risk in people with atopic eczema. Any link could have substantial effect; atopic eczema is common, and fractures have associated morbidity and mortality.

**Objective:**

We sought to examine whether atopic eczema is associated with fracture and whether fracture risk varies with eczema severity.

**Methods:**

We performed a matched cohort study set in primary care (Clinical Practice Research Datalink GOLD 1998-2016) and linked hospital admissions data (Hospital Episode Statistics), including adults (≥18 years old) with atopic eczema matched (by age, sex, general practice, and cohort entry date) with up to 5 individuals without eczema. We estimated hazard ratios (HRs) from stratified Cox regression comparing risk of major osteoporotic (hip, pelvis, spine, wrist, and proximal humerus) fractures individually and any fracture in those with and without atopic eczema.

**Results:**

We identified 526,808 people with atopic eczema and 2,569,030 people without atopic eczema. Those with eczema had increased risk of hip (HR, 1.10; 99% CI, 1.06-1.14), pelvic (HR, 1.10; 99% CI, 1.02-1.19), spinal (HR, 1.18; 99% CI, 1.10-1.27), and wrist (HR, 1.07; 99% CI, 1.03,-1.11) fractures. We found no evidence of increased proximal humeral (HR, 1.06; 99% CI, 0.97-1.15) fracture risk. Fracture risk increased with increasing eczema severity, with the strongest associations in people with severe eczema (compared with those without) for spinal (HR, 2.09; 99% CI, 1.66-2.65), pelvic (HR, 1.66; 99% CI, 1.26-2.20), and hip (HR, 1.50; 99% CI, 1.30-1.74) fractures. Associations persisted after oral glucocorticoid adjustment.

**Conclusions:**

People with atopic eczema have increased fracture risk, particularly major osteoporotic fractures.

## Graphical abstract

**Figure:**
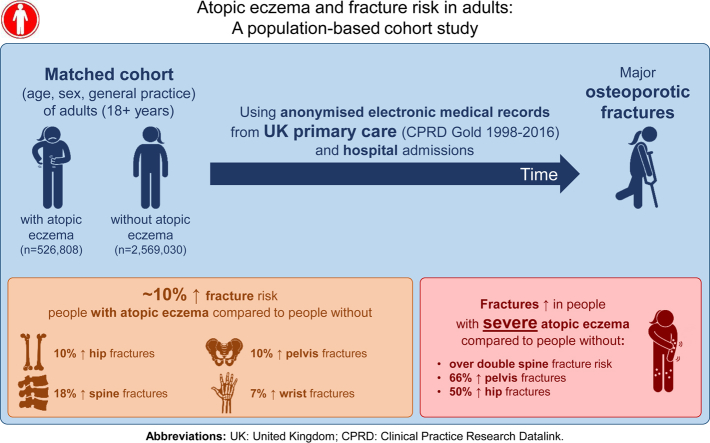

Atopic eczema is a common inflammatory skin disease affecting up to 10% of adults.^1^ Morbidity is substantial, with itching, soreness, stress, impaired sleep, and low self-esteem contributing to reduced quality of life.^2^^,^^3^ Limited evidence suggests that people with atopic eczema might have reduced bone mineral density and might be at increased risk of fractures.^4^, ^5^, ^6^ Reducing fractures is an important public health goal because fractures are associated with increased morbidity and mortality.^7^, ^8^, ^9^ Atopic eczema is common, and therefore any association with fracture could have a major effect.

A 2017 study from Taiwan suggested that osteoporosis is more likely in people with atopic eczema.^10^ Two US-based cross-sectional studies suggest that those with self-reported atopic eczema are at increased risk of self-reported fracture compared with those without eczema.^5^^,^^6^ However, the existing research is limited in its ability to (1) explore the temporal association between atopic eczema and fracture, (2) investigate whether fracture risk increases with increasing atopic eczema severity, and (3) consider important confounders or mediators of the relationship between atopic eczema and fracture risk (including body mass index [BMI], smoking, and oral glucocorticoid use).

We undertook a matched cohort study using electronic health records data to examine whether adults with atopic eczema were at increased risk of major osteoporotic fractures and whether fracture risk varied with increasing eczema severity.

## Methods

### Study design and setting

We undertook a matched cohort study between January 2, 1998, and March 31, 2016, using routinely collected United Kingdom (UK) electronic health records data (primary care data from the Clinical Practice Research Datalink [CPRD Gold] and linked hospital admissions data from Hospital Episode Statistics [HES]). CPRD includes information on diagnoses, treatments, and demographics for approximately 7% of the UK population, with 75% of English general practices linked with HES data.^11^ HES records cover all hospital admissions for National Health Service–funded patients treated in either English National Health Service trusts or by independent providers.^12^

Morbidity code lists for all variables (identifying atopic eczema, fracture outcomes, and covariates) are available for download (https://doi.org/10.17037/DATA.00001156), and we have provided further details regarding variable definitions in the Methods section in this article’s Online Repository at www.jacionline.org.

### Study population

**Fig 1:**
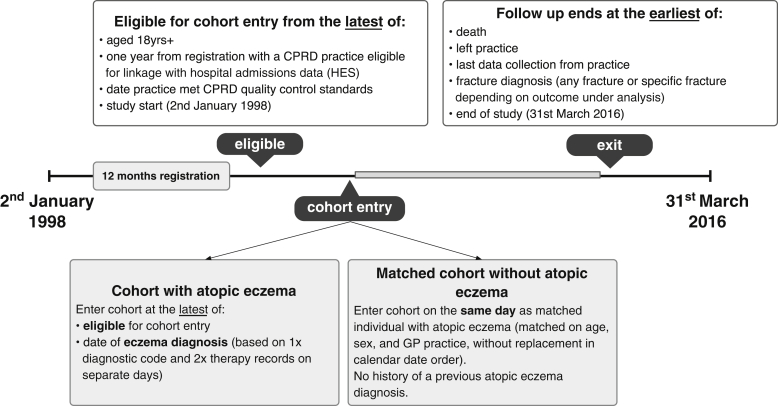
Graphic depiction of the study population. *CPRD*, Clinical Research Practice Datalink; *GP*, general practitioner.

All adults (≥18 years) registered with primary care practices contributing data to the CPRD that met CPRD quality control standards, and with at least 1 year of registration before cohort entry, were eligible for inclusion (Fig 1). We identified a cohort of people with atopic eczema based on a previously validated algorithm^13^ requiring a diagnostic code for eczema and at least 2 records for eczema therapy (see the Methods section in this article’s Online Repository). We randomly selected a matched cohort (without replacement to avoid misleadingly precise SEs potentially introduced by matching with replacement) of up to 5 individuals without eczema to each individual with atopic eczema by age, sex, and general practice (see the Methods section in this article’s Online Repository).

### Fracture outcomes

We identified specific major osteoporotic fractures (of the hip, pelvis, spine, proximal humerus, and wrist) individually and any fracture type recorded by using morbidity coding in primary care (CPRD) or during a hospital admission (HES). We specifically excluded surgical, allograft, autograft, neoplasm-related, or stress-related fractures because they are unlikely to be related to atopic eczema. Individuals were followed until their first fracture diagnosis. For analyses focusing on specific osteoporotic fractures, participants were censored when they experienced the specific fracture of interest (ie, not censored based on history of a different fracture type).

### Covariates

**Fig E1:**
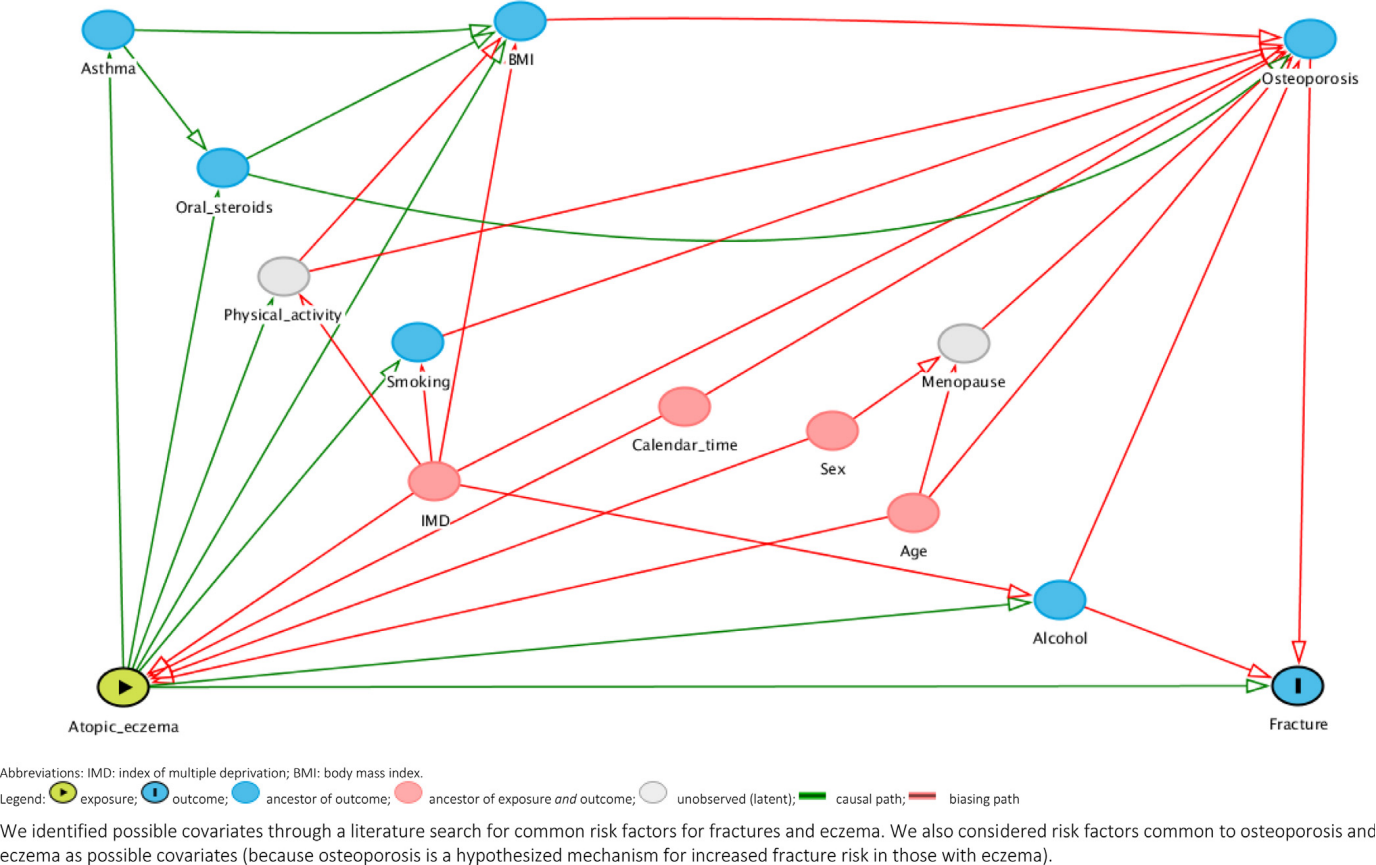
Directed acyclic graph showing the implicitly assumed causal relationship (between atopic eczema and fracture) underlying our adjusted models. We identified possible covariates through a literature search for common risk factors for fractures and eczema. We also considered risk factors common to osteoporosis and eczema as possible covariates (because osteoporosis is a hypothesized mechanism for increased fracture risk in those with eczema).

We used a directed acyclic graph to identify potential confounders, mediators, and colliders of the relationship between atopic eczema and fractures (see Fig E1 in this article’s Online Repository at www.jacionline.org), including age, sex, quintile of the Index of Multiple Deprivation (IMD; proxy for socioeconomic deprivation),^14^ calendar time (1997-2001, 2002-2006, 2007-2011, and 2012-2016 to account for changes in clinical and administrative practices), asthma, BMI, smoking status, harmful alcohol use, and oral glucocorticoid use.

### Statistical analysis

#### Main analysis

We first examined descriptive characteristics for those with and without atopic eczema. We used Cox regression stratified by matched set, with age as the underlying timescale, to estimate hazard ratios (HRs) and their 99% CIs comparing the risk of fracture in those with atopic eczema with the risk in those without atopic eczema. We used 99% CIs throughout to minimize type I error.^15^ A 99% CI is wider than the more standard 95% CIs because if we want to increase the probability that a range of values contains the “true” population parameter (a 99% CI will include the “true” parameter 99% of the time), we need a broader range of containing values.

Initially, we implicitly adjusted for age (due to the underlying timescale) and sex, general practice, and date of cohort entry (due to matching). We then additionally adjusted for calendar period, quintiles of IMD, and time-updated asthma. We then further adjusted for variables that might be on the causal pathway between atopic eczema and fractures (potential mediators): BMI, smoking status, harmful alcohol use, and high-dose oral glucocorticoid exposure. We adjusted for BMI, smoking, and harmful alcohol use in a complete case analysis. We tested the assumption of proportional hazards by using Schöenfeld residual plots.

We tested how robust our findings were by repeating the main analysis after systematically altering aspects of the main study design in a series of sensitivity analyses (see Table E1 in this article’s Online Repository at www.jacionline.org).

#### Secondary analyses

To assess the effect of atopic eczema severity on fractures, we redefined eczema as mild, moderate, or severe and compared fracture risk at each severity level with risk in those without eczema using stratified Cox regression. We defined eczema severity as a time-updated variable, with status changing on the first date that participants met the definitions for moderate or severe eczema. By default, all people with atopic eczema were classified as having mild disease, unless they were (1) prescribed potent topical steroids or calcineurin inhibitors when they were classified as having moderate eczema, or (2) referred to a dermatologist, prescribed a systemic drug (azathioprine, cyclosporine, methotrexate, or mycophenolate mofetil), or had a record for phototherapy (in primary or secondary care) when they were classified as having severe disease. Individuals progressed from mild to moderate eczema at the first record, suggesting moderate disease, and from mild or moderate eczema to severe eczema at the first record, suggesting severe disease.

To test whether age or sex modified the effect of atopic eczema on fracture risk, we stratified the analysis separately by age and sex. We used likelihood ratio tests to test for statistical evidence of effect modification.

We used Stata software (version 15; StataCorp, College Station, Tex) for all analyses. The study protocol was approved by the Independent Scientific Advisory Committee for the CPRD (ISAC protocol no. 16_100RA) and the London School of Hygiene and Tropical Medicine (reference 14645).

#### Patient involvement

Amanda Roberts, our patient representative, helped in developing the research question and the interpretation and writing up of our results.

## Results

**Fig 2:**
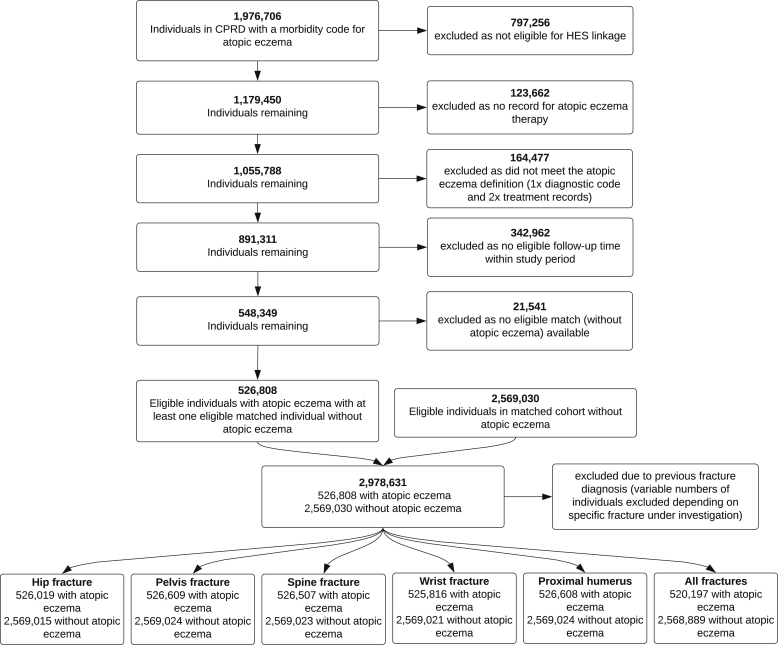
Flow chart illustrating identification of study populations. Note: The number of participants in both the atopic eczema and matched cohorts do not add up to the total number of study participants because participants can contribute follow-up time both with and without atopic eczema.

We identified 526,808 individuals with atopic eczema and 2,569,030 individuals without atopic eczema (Fig 2) who were eligible for inclusion in the study. After excluding those with a previous history of any fracture, we lost 1% (n = 6611) of those with atopic eczema and less than 1% (n = 141) of those without (fewer were excluded for analyses of specific fractures because these analyses only excluded those with a history of the specific fracture). Those with atopic eczema included in the study population for the outcome of any fracture had a median follow-up of 5.0 years (interquartile range, 2.0-9.7 years), and those without had a median follow-up of 4.4 years (interquartile range, 1.7-8.9 years; Table I and see Table E2 in this article’s Online Repository at www.jacionline.org).

**Table I:** Characteristics at cohort entry of the study population∗ for the any fracture analysis

	Individuals with atopic eczema (n = 520,197)	Individuals without atopic eczema (n = 2,568,889)
Total person-years of follow-up	3,118,930	14,146,660
Median follow-up time (IQR)	5.0 (2.0-9.7)	4.4 (1.7-8.9)
Female sex	303,581 (58.4)	1,489,176 (58.0)
Age band at entry (y)		
18-39	245,469 (47.2)	1,217,679 (47.4)
40-49	69,016 (13.3)	351,917 (13.7)
50-59	63,117 (12.1)	328,990 (12.8)
60-69	60,762 (11.7)	303,768 (11.8)
≥70	81,833 (15.7)	366,535 (14.3)
Quintiles of IMD		
5 (most deprived)	74,052 (14.2)	370,174 (14.4)
4	99,223 (19.1)	489,120 (19.0)
3	102,343 (19.7)	508,442 (19.8)
2	119,402 (23.0)	589,283 (22.9)
1 (least deprived)	125,177 (24.1)	611,870 (23.8)
Asthma diagnosis	124,702 (24.0)	318,410 (12.4)
BMI		
Underweight (<18.5 kg/m^2^)	37,297 (7.2)	185,784 (7.2)
Normal weight (18.5-24.9 kg/m^2^)	170,370 (32.8)	828,367 (32.2)
Overweight (25.0-29.9 kg/m^2^)	141,844 (27.3)	667,277 (26.0)
Obese (≥30.0 kg/m^2^)	90,971 (17.5)	393,529 (15.3)
Missing	79,715 (15.3)	494,073 (19.2)
Harmful alcohol use	14,071 (2.7)	57,258 (2.2)
Smoking status		
Nonsmokers	263,336 (50.4)	1,293,912 (50.4)
Current or former smokers	246,022 (46.7)	1,125,564 (43.8)
Missing	13,839 (2.7)	149,413 (5.8)
High-dose oral glucocorticoid prescription†	91,587 (17.6)	191,223 (7.4)

All values are numbers (percentages), unless otherwise stated.

*IQR*, Interquartile range.

∗ Note that the study populations for analyses of specific fracture outcomes are similar to those of the study population displayed above (ie, that for the any fracture analysis), but the specific fracture study populations have fewer exclusions because of previous fractures (because individuals were only excluded from these study populations if they had a history of the specific fracture under investigation). Table E2 shows baseline characteristics for the entire eligible study population before exclusion because of the history of previous fracture and is broadly similar to that above (ie, after exclusion of those with a history of previous fracture).

† Prednisolone equivalent dose of 20 mg/day or more. Further details on variable definitions can be found in the Methods section in this article’s Online Repository.

We saw strong evidence for an association between atopic eczema and increased hip, pelvic, spinal, and wrist fractures after adjusting for calendar period, IMD, and asthma (implicitly adjusted for age, sex, general practice, and date of cohort entry; Table II). There was weaker evidence for an increase in proximal humeral fractures. For any fracture, the HR comparing the risk of fracture in those with and without atopic eczema was 1.10 (99% CI, 1.08-1.12). The greatest increased risk was seen for spinal fracture (HR, 1.18; 99% CI, 1.10-1.27). The unadjusted absolute rate for any fracture in those with atopic eczema was 1428 fractures per 100,000 person-years, an excess of 164 fractures per 100,000 person years compared with matched individuals without eczema.

**Table II:** HRs∗ (99% CIs) comparing fracture risk in those with and without atopic eczema

	No.	Events/person-years at risk	Minimally adjusted†	Adjusted for IMD, asthma, and calendar period‡	Additionally adjusted for potential mediators (BMI, harmful alcohol use, smoking, and oral glucocorticoids‖)§		
			HR∗ (99% CI)	HR∗ (99% CI)	No.	Events/person-years at risk	HR∗ (99% CI)
Hip fractures							
Without atopic eczema	2,569,015	30,592/14,849,062	1 (reference)	1 (reference)	1,839,065	23,041/11,516,122	1 (reference)
With atopic eczema	526,019	7,822/3,326,205	1.11 (1.07-1.16)	1.10 (1.06-1.14)	439,659	6,808/2,965,992	1.06 (1.02-1.11)
Pelvic fractures							
Without atopic eczema	2,569,024	7,337/14,911,177	1 (reference)	1 (reference)	1,839,071	5,590/11,565,470	1 (reference)
With atopic eczema	526,609	1,923/3,343,770	1.12 (1.04-1.21)	1.10 (1.02-1.19)	440,161	1,698/2,981,712	1.06 (0.97-1.16)
Spinal fractures							
Without atopic eczema	2,569,023	8,716/14,904,064	1 (reference)	1 (reference)	1,839,072	7,011/11,559,317	1 (reference)
With atopic eczema	526,507	2,439/3,341,052	1.22 (1.14-1.30)	1.18 (1.10-1.27)	440,066	2,245/2,979,043	1.14 (1.06-1.23)
Wrist fractures							
Without atopic eczema	2,569,021	25,068/14,818,020	1 (reference)	1 (reference)	1,839,071	20,384/11,487,169	1 (reference)
With atopic eczema	525,816	6,210/3,316,871	1.09 (1.05-1.13)	1.07 (1.03-1.11)	439,434	5,641/2,956,472	1.06 (1.01-1.10)
Proximal humeral fractures							
Without atopic eczema	2,569,024	6,428/14,910,404	1 (reference)	1 (reference)	1,839,071	5,590/11,565,470	1 (reference)
With atopic eczema	526,608	1,612/3,343,880	1.08 (0.99-1.17)	1.06 (0.97-1.15)	440,161	1,437/2,981,823	1.03 (0.94-1.13)
Any fracture							
Without atopic eczema	2,568,889	179,471/14,146,660	1 (reference)	1 (reference)	1,838,979	135,663/10,967,230	1 (reference)
With atopic eczema	520,197	44,543/3,118,930	1.13 (1.11-1.14)	1.10 (1.08-1.12)	434,335	38,612/2,779,903	1.07 (1.05-1.09)

∗ Estimated HRs from Cox regression with current age as the underlying timescale stratified by matched set (matched on age at cohort entry, sex, general practice, and date at cohort entry). All models fitted to participants with complete data for all variables included in each model and from valid matched sets, including 1 individual with atopic eczema and at least 1 individual without atopic eczema. All models were implicitly adjusted for sex, date at cohort entry, and practice (because of stratification by matched set) and age (because of underlying timescale).

† Minimally adjusted is defined as implicit adjustment for sex, age, general practice, and date of cohort entry.

‡ Fully adjusted is defined as additionally adjusted for time-updated asthma, IMD, and calendar time.

§ Additionally adjusted for potential mediators is defined as further adjustment for BMI, smoking status, harmful alcohol use, and oral glucocorticoid exposure. Participants were only included if they were in a complete matched set (complete data for 1 individual with atopic eczema and ≥1 individual without atopic eczema).

‖ Time-updated ever-prescribed ≥20 mg/day prednisolone equivalent dose (status changing at first ever prescription).

After further adjusting for potential mediators (BMI, smoking, harmful alcohol use, and high-dose oral glucocorticoid use), the association between atopic eczema and fracture was slightly attenuated (eg, HR for any fracture in those with eczema compared with those without after additionally adjusting for potential mediators of 1.07 [99% CI, 1.05-1.09] compared with HR adjusted for calendar period, IMD, and asthma of 1.10 [99% CI, 1.08-1.12]; see Table E3 in this article’s Online Repository at www.jacionline.org); we saw similar attenuation of effect estimates across all of the major osteoporotic fractures investigated.

### Sensitivity analyses

We saw minimal change in effect estimates for the association between atopic eczema and fracture in most sensitivity analyses (see Table E4 in this article’s Online Repository at www.jacionline.org). However, when those with a previous history of any type of fracture were excluded from analyses of specific fracture outcomes, we saw that (1) for pelvic, wrist, and proximal humeral fractures, the HRs comparing fracture risk in those with and without eczema were reduced and 99% CIs crossed one, but (2) for spine and hip fractures, HRs were attenuated, but the increased risk of fracture remained.

### Atopic eczema severity

**Fig 3:**
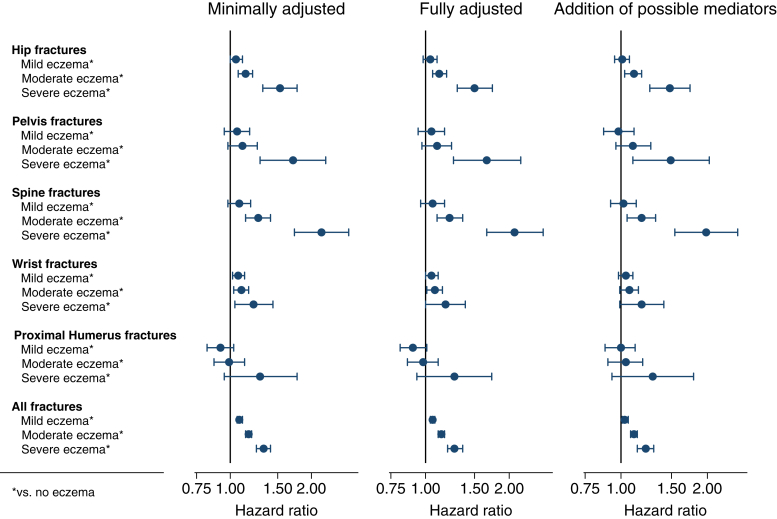
Forest plot showing association (HR [99% CI] compared with those without atopic eczema) between severity of atopic eczema and fracture. *In comparison to those without atopic eczema. *Minimally adjusted* is defined as implicit adjustment for sex, age, general practice, and date of cohort entry. *Fully adjusted* is defined as additional adjustment for time-updated asthma, IMD, and calendar time. *Addition of possible mediators* is defined as further adjustment for BMI, smoking status, harmful alcohol use, and high-dose oral glucocorticoid use. Participants were only included if they were in a complete matched set (complete data for 1 individual with atopic eczema and ≥1 individual without atopic eczema).

Risk of fracture increased with increasing atopic eczema severity (Fig 3 and see Table E5 in this article’s Online Repository at www.jacionline.org). For example, there was a 6% increase in the risk of spinal fracture in people with mild eczema (HR, 1.06; 99% CI, 0.96-1.17), a 22% increase in those with moderate eczema (HR, 1.22; 99% CI, 1.10-1.36), and a 109% increase in those with severe eczema (HR, 2.09; 99% CI, 1.66-2.65). The greatest magnitude of increased risk was for spinal fracture in those with severe eczema (HR, 2.09; 99% CI, 1.66-2.65), followed by pelvic (HR, 1.66; 99% CI, 1.26-2.20) and hip (HR, 1.50; 99% CI, 1.30-1.74) fractures.

### Effect modification

We found varying evidence depending on specific fracture outcomes for age modifying the effect of atopic eczema on fracture risk (see Table E6 in this article’s Online Repository at www.jacionline.org), with some statistical evidence that age modified the effect of atopic eczema on spinal (*P* = .0007) and hip (*P* = .0043) fractures, but 99% CIs for stratum-specific estimates overlapped.

Similarly, evidence for sex modifying the effect of atopic eczema on fracture risk varied depending on specific fracture outcome, with some evidence of a slightly greater risk of hip, wrist, and any fracture associated with eczema in men than in women; however, 99% CIs for stratum-specific estimates overlapped.

## Discussion

We found that atopic eczema was associated with an increase in fracture risk, particularly among people with severe eczema. The increased risk was most pronounced for major osteoporotic fractures; spinal fracture risk more than doubled in those with severe eczema compared with those without atopic eczema, whereas hip fracture rates increased by 50% and pelvic fracture rates were increased by 66%.

### Strengths and weaknesses

Our study is the largest to date examining the relationship between atopic eczema and fractures and the first using primary care data. Our results are likely to be representative of the general population of England. The algorithm used to identify atopic eczema has been validated in a similar UK primary care database with a positive predictive value of 82%.^13^ Although some people with atopic eczema might have been wrongly identified as not having atopic eczema, and some without eczema might have been wrongly identified as having atopic eczema, this misclassification would bias the effect estimate toward the null, meaning that our estimates might be cautious estimates of the true increase in fracture risk associated with atopic eczema. The strict definition of atopic eczema we used (requiring ≥1 diagnostic code and ≥2 treatment records) improved precision but might mean some people with eczema were missed. However, after broadening our eczema definition to include anyone with an eczema diagnosis (not requiring 2 records of eczema therapy), we saw similar results.

We did not exclude participants with a history of any type of fracture in analyses of specific fracture outcomes. Previous fractures affect the risk of future fractures, which might have affected our results but is unlikely to result in bias.^16^ After repeating the main analysis excluding individuals with a history of any fracture, we saw a similar increased risk of hip and spinal fractures for those with eczema compared with those without, but HRs for pelvic wrist, and proximal humeral fractures were reduced, and their 99% CIs crossed 1.

It is difficult to capture the exact onset of relapsing conditions, such as atopic eczema, using electronic health data. Therefore we used the recommended dynamic cohort approach, including individuals with both “new” and existing atopic eczema.^17^ Inclusion of prevalent atopic eczema maximized our sample size, and a sensitivity analysis limited to individuals with newly active atopic eczema and their matched counterparts showed broadly similar results.

Individuals included in the atopic eczema cohort were identified based on primary care prescriptions and morbidity coding. Therefore it is possible that our study selectively included people more likely to consult their general practitioner with atopic eczema than those without. This has implications for identification of all variables used in the study (eczema exposure and covariates and, to a lesser extent, fracture outcomes) because those more likely to consult a health care practitioner are more likely to have data recorded capturing these variables. Most fractures are painful and not usually missed in primary or secondary care, and therefore it is likely that we captured the majority of fracture outcomes. However, spinal fractures might not be detected, and people might not know that they have a fracture.^18^ Therefore the high risk of spinal fracture in those with severe eczema compared to those without (HR, 2.09; 99% CI, 1.66-2.65) might be due, at least in part, to bias in fracture detection. Those with severe eczema are likely to consult a physician more frequently and might be more likely to receive a diagnosis of a spinal fracture than those who consult their general practitioner less frequently. However, we saw minimal difference in effect estimates from sensitivity analyses limited to those who had attended their general practice in the year before cohort entry (ie, practice attenders), suggesting that this did not introduce substantial bias.

There is some evidence that topical steroids may be absorbed systemically, suggesting that topical steroid use might contribute to increased fracture risk in those with atopic eczema treated with topical steroids.^19^ It is unclear whether sufficient quantities to increase fracture risk can be absorbed; absorption is dependent on skin integrity, steroid dosage, and adherence, all of which are difficult to capture in routine data. Furthermore, topical steroid use is likely to be confounded by indication and eczema severity. Our analyses, including high-dose oral glucocorticoid use, demonstrated only a small attenuation of the effect of atopic eczema on fracture risk, suggesting it would be unlikely that lower doses of steroids absorbed through the skin would explain the association; however, additional research is needed to directly address this question.

Our study is limited because data are collected as part of routine care rather than specifically for research. We could not account for some potential confounders or mediators of the association between eczema and fractures (eg, physical activity levels, vitamin D levels, food allergy or intolerance, malnourishment, eating disorders, antihistamines, or fatigue) in our analyses because these data are either not collected systematically or not collected robustly (although many are captured indirectly through BMI). Clinical experience suggests that restrictive diets are common in people with atopic eczema.^20^ Furthermore, those with complete data on confounders, such as vitamin D, food allergy, and eating disorders, are likely to be systematically different from those with incomplete data. It is possible that the anticholinergic effect of antihistamines (available over the counter and therefore not captured robustly in prescribing data) used to treat allergic rhinitis (associated with eczema)^21^ or fatigue (caused by itch from eczema disturbing sleep) could increase risk of fall and subsequent fracture. As a result, residual confounding might contribute to our findings.

Unlike other fracture types, we saw no evidence for an association between atopic eczema and proximal humeral fractures. One explanation might be the accuracy of primary care coding for proximal humeral fractures in comparison with our other specific fracture outcomes (eg, proximal humeral fractures might be coded nonspecifically as “arm fracture”). The effect of coding granularity is demonstrated by our sensitivity analysis limited to specific proximal humeral fracture codes (our main analysis included more ambiguous codes for “shoulder” or “upper arm” fractures, see Table E4).

### Comparison with other studies

Atopic eczema is associated with chronic inflammation, which has been associated with osteoporosis and fractures in people with other inflammatory diseases, including rheumatoid arthritis and inflammatory bowel disease.^22^, ^23^, ^24^, ^25^ Limited previous studies from the United States and Taiwan suggest that those with atopic eczema are at greater risk of osteoporosis or fracture. These findings are consistent with ours, but these previous studies lacked data on important confounders.^4^, ^5^, ^6^^,^^10^^,^^26^ Our study addresses many of the shortcomings of the previous studies because we were able to capture physicians’ diagnoses of both atopic eczema (by using a validated algorithm) and fracture, as well as potential confounders and mediators of the relationship. Therefore, unlike previous studies, we were able to determine the temporality of the relationship between atopic eczema and fracture, adjust for important confounders, and consider potential mediators of the relationship. In addition to demonstrating an increased risk of any fracture type and of specific osteoporosis-related fractures, our results further suggest that increased fracture risk has a dose-response relationship with atopic eczema severity.

Our study shows that the increased risk of low bone density and osteoporosis in people with atopic eczema demonstrated in previous studies^4^^,^^10^^,^^26^^,^^27^ might translate into increased fracture risk. We were unable to examine bone density (thorough dual-energy x-ray absorptiometry) or osteoporosis directly because this information is not consistently captured in routine data. However, our observation of increased fracture risk in those with atopic eczema highlights fracture as an important adverse health outcome in people with severe eczema.

### Implications for clinical practice

The UK National Osteoporosis Guideline Group^28^ and the US Preventative Services Task Force^29^ recommend the Fracture Risk Assessment Tool algorithm to assess fracture risk.^30^ English^31^ and Scottish^32^ national guidelines also recommend the QFracture algorithm, which uses primary care data to estimate osteoporotic fracture risk.^33^ The Fracture Risk Assessment Tool and QFracture algorithms account for many factors that increase fracture risk, including secondary osteoporosis (or disorders strongly associated with osteoporosis, such as premature menopause and malnutrition), but atopic eczema is not considered. Our findings suggest that atopic eczema should be added to the factors considered in fracture prediction, and future studies should explore whether targeted screening and intervention would benefit individuals with atopic eczema. Our findings also support the recent statement from the International Eczema Council regarding avoidance of systemic steroids for atopic eczema.^34^ Additional research should focus on determining possible biological mechanisms linking atopic eczema to decreased bone density.

### Conclusion

We have shown that atopic eczema is associated with an increased fracture risk. The substantial increase in the risk of spinal, hip, and pelvic fractures seen in those with severe atopic eczema is particularly concerning (more than double the risk of spinal fracture, 66% increased risk of pelvic fracture, and 50% increased risk of hip fracture) given the high morbidity and mortality associated with these fractures. Our results suggest that bone density screening guidelines should consider including individuals with more severe atopic eczema to prevent fractures, improve long-term quality of life, and reduce fracture-related health care costs.

**Clinical implications**

People with more severe atopic eczema might benefit from targeted bone density testing and strategies for fracture prevention.

## Figures and Tables

**Table E1:** Sensitivity analyses

Sensitivity analysis	Justification
1. Analyses for specific fracture outcomes were repeated, excluding subjects with a history of *any* prior fracture.	The main analysis approach, in which we only excluded those with a previous history of the specific fracture outcome under investigation, assumes that a fracture will only affect subsequent fracture probability in the same bone. However, a fracture in one bone can affect fracture risk in another bone. To test this, we repeated the main analysis after additionally excluding those with a history of any previous fracture.
2. Main analysis was repeated and restricted to those who registered with a general practice after 2006 and was additionally adjusted for ethnicity.	Ethnicity can affect fracture risk, but ethnicity was not routinely and accurately coded in the CPRD until 2006 onward, when its recording was incentivized in the quality and outcomes framework.
3. Main analysis was repeated on a redefined cohort in which the unexposed pool of individuals included those with a diagnosis of atopic eczema but without 2 records of treatment (did not fully meet the diagnostic criteria in the algorithm), and subjects remained in the unexposed pool until they fully met the criteria of the algorithm. This cohort was matched separately to the main analysis cohort.	To explore the sensitivity of the results to the definition of the exposure
4. Main analysis was repeated on a redefined cohort in which the exposed cohort included all individuals with an atopic eczema diagnostic code (did not require treatment codes). This cohort was matched separately to the main analysis cohort.	To explore the sensitivity of the results to the definition of the exposure
5. Main analysis was repeated after restricting to those with newly active atopic eczema (ie, first eczema diagnosis recorded during valid follow-up) and their matched counterparts.	To ensure that covariates measured at entry precede atopic eczema onset and are therefore less likely to lie on the causal pathway between atopic eczema and fracture outcomes
6. Main analysis was repeated, restricting the cohort to those who had visited their general practice in the year before entering the cohort.	To exclude practice nonattenders
7. Proximal humeral fracture analysis was repeated by using a stricter definition of proximal humeral fractures (excluding fractures coded as shoulder or upper arm fractures).	To assess the effect of proximal humeral fracture outcome definition in primary care given that there is no previously validated electronic health record based definition for proximal humeral fractures

Note: For simplicity, the any fracture outcome was used for all sensitivity analyses, except those testing the definition of specific fracture outcomes.

**Table E2:** Characteristics at cohort entry of all individuals eligible for inclusion in the study population (before excluding individuals with a history of fracture)

	With atopic eczema (n = 526,808)	Without atopic eczema (n = 2,569,030)
Total person-years of follow-up	3,248,048	14,932,306
Median follow-up time (IQR)	5.0 (2.0-9.6)	4.4 (1.7-8.9)
Female sex	308,071 (58.5)	1,489,261 (58.0)
Age band at entry (y)		
18-39	246,596 (46.8)	1,217,722 (47.4)
40-49	69,696 (13.2)	351,927 (13.7)
50-59	63,943 (12.1)	329,007 (12.8)
60-69	61,902 (11.8)	303,790 (11.8)
≥70	84,671 (16.1)	366,584 (14.3)
Quintiles of IMD		
5 (most deprived)	74,980 (14.2)	370,200 (14.4)
4	100,430 (19.1)	489,144 (19.0)
3	103,646 (19.7)	508,469 (19.8)
2	120,946 (23.0)	589,313 (22.9)
1 (least deprived)	126,806 (24.1)	611,904 (23.8)
Asthma diagnosis	126,180 (24.0)	318,433 (12.4)
BMI		
Underweight (<18.5 kg/m^2^)	37,756 (7.2)	185,784 (7.2)
Normal weight (18.5-24.9 kg/m^2^)	172,446 (32.7)	828,367 (32.2)
Overweight (25.0-29.9 kg/m^2^)	143,919 (27.3)	667,277 (26.0)
Obese (≥30.0 kg/m^2^)	92,507 (17.7)	393,529 (15.3)
Missing	80,180 (15.2)	494,073 (19.2)
Harmful alcohol use	14,438 (2.7)	57,268 (2.2)
Smoking status		
Nonsmokers	266,134 (50.5)	1,293,983 (50.4)
Current or former smokers	246,782 (46.8)	1,125,627 (43.8)
Missing	13,892 (2.64)	149,420 (5.82)
High-dose oral glucocorticoid prescription∗	93,443 (17.7)	191,246 (7.4)

All values are shown as numbers (percentages), unless otherwise stated.

*IQR*, Interquartile range.

∗ Prednisolone equivalent dose of 20 mg/day or more. Further details on variable definitions can be found in the Methods section in this article’s Online Repository.

**Table E3:** HRs (99% CIs) for association between all variables included in regression models adjusting for potential mediators and any fracture

	HR∗ (99% CI)	
	Adjusted for IMD, calendar time, asthma, harmful alcohol use, smoking, and BMI	Additionally adjusted for high-dose oral glucocorticoid use
Atopic eczema		
Without atopic eczema	1.0 (reference)	1.0 (reference)
With atopic eczema	1.08 (1.06-1.10)	1.07 (1.05-1.09)
Calendar time		
1997-2001	1.0 (reference)	1.0 (reference)
2002-2006	0.96 (0.83-1.11)	0.96 (0.83-1.11)
2007-2011	0.97 (0.82-1.15)	0.97 (0.82-1.14)
2012-2016	0.98 (0.82-1.17)	0.97 (0.81-1.16)
IMD		
1 (least deprived)	1.0 (reference)	1.0 (reference)
2	1.05 (1.02-1.07)	1.05 (1.02-1.07)
3	1.08 (1.05-1.11)	1.08 (1.05-1.11)
4	1.13 (1.10-1.17)	1.13 (1.10-1.16)
5 (most deprived)	1.19 (1.15-1.23)	1.19 (1.15-1.23)
Asthma		
No asthma	1.0 (reference)	1.0 (reference)
Asthma	1.25 (1.23-1.28)	1.18 (1.15-1.20)
Harmful alcohol use		
None documented	1.0 (reference)	1.0 (reference)
Harmful use	1.96 (1.88-2.06)	1.96 (1.87-2.05)
Smoking		
Never-smoker	1.0 (reference)	1.0 (reference)
Current or former	1.18 (1.16-1.20)	1.17 (1.15-1.19)
BMI (kg/m^2^)		
Underweight (<18.5 kg/m^2^)	1.14 (1.10-1.17)	1.14 (1.10-1.17)
Normal weight (18.5-24.9 kg/m^2^)	1.0 (reference)	1.0 (reference)
Overweight (25.0-29.9 kg/m^2^)	0.92 (0.91-0.94)	0.92 (0.91-0.94)
Obese (≥30.0 kg/m^2^)	0.86 (0.85-0.88)	0.86 (0.84-0.88)
High-dose oral glucocorticoid use		
<20 mg PED/day	NA	1.0 (reference)
≥20 mg PED/day	NA	1.20 (1.17-1.23)

*NA*, Not available; *PED*, prednisolone equivalent dose.

∗ Estimated HRs from Cox regression with current age as the underlying timescale stratified by matched set (matched on age at cohort entry, sex, general practice, and date at cohort entry). All models fitted to individuals with complete data for all variables included in each model and from valid matched sets, including 1 individual with atopic eczema and at least 1 individual without atopic eczema. Adjustments were made for calendar time, IMD, asthma, harmful alcohol use, smoking and BMI, and implicitly adjusted for sex, date at cohort entry, and practice (because of stratification by matched set) and age (because of underlying timescale).

**Table E4:** HRs (99% CIs) for association between atopic eczema and fractures for main and sensitivity analyses

Analysis	No. of individuals	Person-years at risk	No. of fracture events	HR∗ (99% CI) for fracture risk in those with atopic eczema compared with those without	
				Adjusted for potential confounders†	Additionally adjusted for potential mediators‡
**Any fracture**					
Main analysis	2,978,407	17,265,591	224,016	1.10 (1.08-1.12)	1.08 (1.06-1.10)
Additionally adjusted for ethnicity and restricted to those entering the cohort after 2006	640,317	2,105,275	18,576	1.06 (0.98-1.15)	1.04 (0.95-1.15)
Redefined cohort 1 (unexposed pool of subjects included those with a diagnosis of atopic eczema but without 2 records of treatment)	2,912,218	16,985,396	221,985	1.10 (1.08-1.11)	1.07 (1.05-1.09)
Redefined cohort 2 (included all patients with an atopic eczema diagnosis code)	3,509,617	21,050,236	260,151	1.09 (1.07-1.10)	1.06 (1.04-1.09)
Restricting the cohort to those with newly diagnosed eczema and their matched counterparts	324,119	1,580,515	23,009	1.11 (1.05-1.16)	1.09 (1.04-1.16)
Restricting the cohort to those who have consulted their general practitioner at least once in the year before cohort entry	2,375,594	13,937,539	192,760	1.05 (1.04-1.07)	1.04 (1.02-1.06)
**Hip fractures**					
Main analysis	2,978,603	18,175,267	38,414	1.10 (1.06-1.14)	1.07 (1.03-1.12)
Hip fractures excluding those a with history of any other fracture	2,634,138	15,078,531	27,821	1.10 (1.06-1.16)	1.09 (1.03-1.14)
**Pelvic fractures**					
Main analysis	2,978,623	18,254,948	9,260	1.10 (1.02-1.19)	1.08 (0.99-1.18)
Pelvic fractures excluding those with history of any other fracture	2,740,367	16,216,496	7,422	1.07 (0.97-1.17)	1.04 (0.94-1.15)
**Spinal fractures**					
Main analysis	2,978,621	18,245,116	11,155	1.18 (1.10-1.27)	1.17 (1.09-1.26)
Spinal fractures excluding those with a history of any other fracture	2,742,247	16,227,594	9,180	1.09 (1.01-1.18)	1.09 (1.00-1.19)
**Wrist fractures**					
Main analysis	2,978,611	18,134,891	31,278	1.07 (1.03-1.11)	1.06 (1.02-1.11)
Wrist fractures excluding those with a history of any other fracture	2,759,460	16,294,062	26,329	1.00 (0.93-1.07)	1.01 (0.94-1.08)
**Proximal humeral fracture**					
Main analysis	2,978,624	18,254,284	8,040	1.06 (0.97-1.15)	1.05 (0.94-1.15)
Proximal humeral fractures excluding those with a history of any other fracture	2,738,861	16,206,684	6,402	0.93 (0.84-1.03)	0.92 (0.83-1.02)
Restricting to more specific codes for proximal humeral fracture	3,095,632	18,255,196	8,895	0.95 (0.88-1.03)	0.97 (0.89-1.06)

∗ Estimated HRs from Cox regression with current age as the underlying timescale stratified by matched set (matched on age at cohort entry, sex, general practice, and date at cohort entry). All models fitted to individuals with complete data for all variables included in each model and from valid matched sets, including 1 individual with atopic eczema and at least 1 individual without atopic eczema. All models implicitly adjusted for sex, date at cohort entry, and practice (because of stratification by matched set) and age (because of underlying timescale).

† Additionally adjusted for potential confounders: time-updated asthma, IMD, and calendar time.

‡ Further adjusted for possible mediators of the relationship between fractures and atopic eczema: BMI, smoking status, harmful alcohol use, and high-dose oral glucocorticoid use.

**Table E5:** HRs (99% CIs) for association between atopic eczema disease severity and fracture for all fracture types

	Number	Events/person-years at risk	Minimally adjusted	Adjusted for IMD, asthma, and calendar period	Additionally adjusted for potential mediators (BMI, harmful alcohol use, smoking, and oral glucocorticoids)		
			HR∗ (99% CI)	HR∗ (99% CI)	No.	Events/person-years at risk	HR∗ (99% CI)
Hip fractures							
Without atopic eczema	2,569,015	30,592/14,849,062	1.0 (reference)	1.0 (reference)	1,839,065	23,041/11,516,122	1.0 (reference)
Mild atopic eczema	392,499	3,948/2,029,502	1.05 (1.0-1.11)	1.04 (0.98-1.10)	325,586	3,392/1,781,494	1.01 (0.95-1.07)
Moderate atopic eczema	186,158	3,256/1,110,516	1.14 (1.07-1.21)	1.12 (1.06-1.19)	163,921	2,864/1,015,951	1.11 (1.03-1.18)
Severe atopic eczema	34,378	618/186,187	1.53 (1.32-1.77)	1.50 (1.30-1.74)	29,515	552/186,547	1.48 (1.26-1.74)
Pelvic fractures							
Without atopic eczema	2,569,024	7,337/14,911,177	1.0 (reference)	1.0 (reference)	1,839,071	5,590/11,565,470	1.0 (reference)
Mild atopic eczema	392,845	1,007/2,037,740	1.06 (0.95-1.18)	1.05 (0.94-1.17)	325,913	880/1,788,809	0.98 (0.87-1.11)
Moderate atopic eczema	186,649	752/611,986	1.11 (0.98-1.26)	1.10 (0.97-1.24)	164,349	672/1,022,940	1.10 (0.96-1.27)
Severe atopic eczema	34,577	164/103,941	1.71 (1.29-2.26)	1.66 (1.26-2.20)	29,664	146/169,964	1.49 (1.10-2.03)
Spinal fractures							
Without atopic eczema	2,569,023	8,716/14,904,064	1.0 (reference)	1.0 (reference)	1,839,072	7,011/11,559,317	1.0 (reference)
Mild atopic eczema	392,773	1,203/2,036,459	1.08 (0.98-1.19)	1.06 (0.96-1.17)	325,852	1,087/1,787,605	1.02 (0.92-1.13)
Moderate atopic eczema	186,586	993/1,117,173	1.27 (1.14-1.41)	1.22 (1.10-1.36)	164,286	930/1,021,841	1.18 (1.05-1.32)
Severe atopic eczema	34,533	243/187,419	2.18 (1.73-2.75)	2.09 (1.66-2.65)	29,639	228/169,598	1.98 (1.54-2.55)
Wrist fractures							
Without atopic eczema	2,569,021	25,068/14,818,020	1.0 (reference)	1.0 (reference)	1,839,071	20,384/11,487,169	1.0 (reference)
Mild atopic eczema	392,260	3,520/2,023,035	1.07 (1.02-1.13)	1.05 (1.00-1.11)	325,379	3,162/1,775,172	1.04 (0.98-1.10)
Moderate atopic eczema	186,034	2,284/1,107,864	1.10 (1.03-1.17)	1.08 (1.01-1.15)	163,770	2,105/1,013,070	1.07 (0.99-1.15)
Severe atopic eczema	34,432	406/185,970	1.22 (1.04-1.44)	1.18 (1.00-1.39)	29,546	374/168,230	1.18 (0.99-1.41)
Proximal humeral fractures							
Without atopic eczema	2,569,024	7,337/14,911,177	1.0 (reference)	1.0 (reference)	1,839,075	5,218/11,563,988	1.0 (reference)
Mild atopic eczema	392,846	813/2,037,888	0.92 (0.82-1.03)	0.90 (0.81-1.01)	325,933	773/1,788,900	1.0 (0.88-1.12)
Moderate atopic eczema	186,657	629/1,118,300	0.99 (0.87-1.13)	0.98 (0.86-1.11)	164,354	602/1,022,750	1.04 (0.90-1.19)
Severe atopic eczema	34,560	116/103,957	1.29 (0.95-1.77)	1.27 (0.93-1.73)	29,675	112/170,017	1.29 (0.93-1.79)
Any fracture							
Without atopic eczema	2,568,889	179,471/14,146,660	1.0 (reference)	1.0 (reference)	1,838,979	135,663/10,967,230	1.0 (reference)
Mild atopic eczema	387,988	25,743/1,914,235	1.08 (1.06-1.11)	1.06 (1.04-1.08)	321,523	21,755/1,680,248	1.03 (1.01-1.06)
Moderate atopic eczema	181,742	15,883/1,033,021	1.17 (1.14-1.20)	1.14 (1.11-1.17)	159,818	14,263/944,385	1.11 (1.08-1.14)
Severe atopic eczema	33190	2,919/171,673	1.33 (1.25-1.41)	1.27 (1.20-1.36)	28,428	2,594/155,271)	1.22 (1.14-1.30)

∗ Estimated HRs from Cox regression with current age as the underlying timescale stratified by matched set (matched on age at cohort entry, sex, general practice, and date at cohort entry). All models fitted to individuals with complete data for all variables included in each model and from valid matched sets, including 1 individual with atopic eczema and at least 1 individual without atopic eczema. All models implicitly adjusted for sex, date at cohort entry, and practice (because of stratification by matched set) and age (because of underlying timescale).

**Table E6:** Association (HRs [99% CIs]) between atopic eczema and fracture stratified separately by age group and sex

	HR (99% CI) comparing the risk of fracture in those with and without atopic eczema∗					
	Hip fractures	Spinal fractures	Pelvic fractures	Wrist fractures	Proximal humeral fractures	Any fracture
Age bands (y)	*P* = .0043†	*P* = .0007†	*P* = .6310†	*P* = .4298†	*P* = .4996†	*P* = .2279†
18-49	1.30 (1.11-1.53)	1.02 (0.87-1.20)	1.10 (0.89-1.35)	1.13 (1.04-1.23)	0.99 (0.78-1.25)	1.10 (1.07-1.13)
50-69	1.19 (1.08-1.31)	1.21 (1.06-1.38)	1.19 (0.99-1.43)	1.07 (1.00-1.15)	1.10 (0.95-1.28)	1.12 (1.08-1.15)
≥70	1.09 (1.05-1.14)	1.27 (1.16-1.38)	1.12 (1.03-1.23)	1.07 (1.00-1.13)	1.08 (0.98-1.21)	1.12 (1.09-1.15)
Sex	*P* = .0084†	*P* = .7093†	*P* = .9577†	*P* = .0079†	*P* = .5025†	*P* = .0004†
Male	1.17 (1.08-1.26)	1.17 (1.05-1.31)	1.10 (0.94-1.29)	1.15 (1.06-1.26)	1.09 (0.93-1.28)	1.13 (1.10-1.16)
Female	1.08 (1.03-1.13)	1.19 (1.09-1.30)	1.10 (1.01-1.21)	1.05 (1.00-1.10)	1.05 (0.95-1.15)	1.08 (1.06-1.10)

∗ HRs from stratified Cox regression implicitly adjusted for age, sex, general practice, and date of cohort entry and explicitly adjusted for time-updated asthma, IMD, and calendar time.

† *P* values from a likelihood ratio test comparing Cox regression models with and without interaction terms (age or sex as appropriate).

## Methods

### Variable definitions

We identified atopic eczema using a validated algorithm based on a record of at least 1 diagnostic morbidity code (recorded in either primary or secondary care) and at least 2 records (on separate days) for eczema therapy (recorded in primary care using Read morbidity codes or prescription data).^E1^ Eczema therapy included electronic health records for (1) phototherapy (primary care morbidity coding or hospital data) and (2) primary care prescriptions for topical emollients, corticosteroids or calcineurin inhibitors, or oral corticosteroids, azathioprine, methotrexate, cyclosporine, or mycophenolate.

We identified phototherapy using Read coding in primary care and Office of Population Censuses and Surveys (OPCS) procedure coding in secondary care. We identified systemic treatment of atopic eczema using primary care prescribing records. Finally, we identified hospital admissions for atopic eczema as any hospitalization in which an International Classification of Diseases, Tenth Revision, code for eczema was recorded in the primary diagnostic position of any episode of an admission.

The IMD is a measure of relative deprivation between small geographic regions in England.^E2^ IMD is based on income, employment, education, health, crime, barriers to health and services, and living environment. For this study, we categorized patient-level IMD data into quintiles. We used the 2007 version of IMD data (IMD data are available for the years 2004, 2007, 2010, and 2015) and chose this as the midpoint of the study (January 1998 to March 2016). However, where patient-level data were unavailable, we used practice-level data from England (because of the requirement for HES linkage, we used English data only) in 2010 (the closest available English practice-level data to 2007; ie, the version of patient-level data used).

We defined participants as asthmatic from their first record of a diagnostic code for asthma. Asthma is a common comorbid condition in people with eczema. Asthma is often treated with inhaled corticosteroids, which have been associated with increased risk of fracture and lower bone density at high doses. There is no clear evidence of an increased risk of fracture at lower doses of inhaled corticosteroids. However, data on inhaled steroid use might not be captured robustly using CPRD data because a prescription does not mean that an individual has used a medication (particularly for drugs that are prescribed for use on an “as-needed” basis), and therefore we used asthma status as a proxy for inhaled steroid exposure. Asthma status was time updated at the first diagnosis of asthma (recorded in CPRD or HES), and individuals were considered to have asthma from that date onward.

We defined harmful alcohol use based on morbidity coding in primary care suggesting harmful or heavy alcohol use (including alcohol dependency codes and codes related to physical/psychological harm related to alcohol use) or a prescription for drugs used to maintain abstinence (acamprosate, disulfiram, or nalmefene). We defined individuals as harmful alcohol users on the date of the first record of a relevant morbidity code or prescription.

We defined high-dose oral glucocorticoid use as a dose of 20 mg/day or more prednisolone equivalent dose. Oral glucocorticoids can be used to treat eczema (and associated diseases, such as asthma) and are known to reduce bone density (potentially leading to osteoporosis and increasing fracture risk) and therefore might be on the causal pathway between eczema and fractures.E3, E4, E5 Currently, oral glucocorticoids are rarely used to treat eczema but can be used for severe flare-ups, and their use might have been more common in the past.E6, E7 We defined individuals as ever or never exposed to high-dose oral glucocorticoid, with status changing on the date of their first recorded prescription of 20 mg/day or more prednisolone equivalent dose. We identified prescriptions for oral corticosteroids with glucocorticoid activity (prednisolone, betamethasone, deflazacort, dexamethasone, hydrocortisone, methylprednisolone, prednisone, triamcinolone, and cortisone) and converted the daily dose prescribed to prednisolone equivalent dose.

BMI and smoking status were defined pragmatically by using the status recorded closest to cohort entry date (with records: within the year before to 1 month after cohort entry date was regarded as best, from 1 month to 1 year after cohort entry was regarded as second best, the most recent record prior to 12 months before cohort entry date was regarded as third best, and within a year from cohort entry date was regarded as worst). BMI was classified into 4 categories: underweight (BMI, <18.5 kg/m^2^), normal weight (BMI, 18.5-24.9 kg/m^2^), overweight (BMI, 25.0-29.9 kg/m^2^), and obese (BMI, ≥30.0 kg/m^2^), according to World Health Organization categories. Smoking status was categorized as current or former smoker versus never smoker.

Morbidity code lists used to define variables are available for download (https://doi.org/10.17037/DATA.00001156).

### Matched cohort

We randomly selected a matched cohort (without replacement) of up to 5 individuals without eczema to each individual with atopic eczema by age, sex, and general practice. We allowed a 15-year age difference for matching to maximize the possibility of successfully matching people with atopic eczema and therefore maximize the generalizability of our cohort. We accounted for the wide 15-year age-matching window by finely adjusting for age as the underlying timescale in all analyses. Matched participants without atopic eczema entered the cohort on the same date as their eczema-exposed counterparts. Matches were assigned to eczema-exposed subjects in calendar date order, and therefore individuals without eczema were assigned to the eczema-exposed group, with the earliest cohort entry date first (to avoid time-related bias). We required that individuals included in the comparison cohort had no history of an atopic eczema diagnosis before cohort entry. However, people with atopic eczema who had valid follow-up time before their eczema diagnosis were eligible for inclusion in the matched comparison cohort until their first record of a diagnostic code for eczema.
